# Dietary Fat-Accelerating Leptin Signaling Promotes Protumorigenic Gastric Environment in Mice

**DOI:** 10.3390/nu11092127

**Published:** 2019-09-06

**Authors:** Seiya Arita, Takumi Ogawa, Yuta Murakami, Yuta Kinoshita, Masaharu Okazaki, Kyoko Inagaki-Ohara

**Affiliations:** Division of Host Defense, Department of Life Sciences, Faculty of Life and Environmental Sciences, Prefectural University of Hiroshima, 5562 Nanatsuka, Shobara, Hiroshima 727-0023, Japan

**Keywords:** high-fat, leptin, stomach, microbiota, protumorigenesis

## Abstract

Excess of fat intake leads to obesity and causes a variety of metabolic diseases and cancer. We previously demonstrated that high-lard diet induces intestinal metaplasia, a precancerous lesion of the stomach mediated by leptin signaling. This study aims to investigate which kinds of dietary fat cause the intestinal metaplasia onset. We fed eight kinds of high-fat diets (HFDs) of animal or plant origin to mice evaluated their effect on gastric pathogenesis. Five types of dietary fat were divided according to their observed effects: Obese with high metaplasia (group I; beef tallow, lard, and hydrogenated coconut oil), non-obese with high metaplasia (group II; linseed oil), obese without metaplasia (group III; corn oil and olive oil), non-obese without metaplasia (group IV, soybean oil) and lean without metaplasia (group V; cocoa butter). The group I and II diets induced leptin, phosphorylated leptin receptor (ObR), signal transducer and activator 3 (STAT3), and increased intracellular β-catenin accumulation in the stomach. Moreover, mice fed these HFDs with 1-methyl-3-nitro-1-nitrosoguanidine (MNNG), a gastric carcinogen, and further accelerated dysplasia in the stomach. *Lactobacillus* occupancy in the stomach increased in all HFDs except hydrogenated coconut oil. Our findings suggest that HFDs inducing leptin signaling accelerate the enhancement of protumorigenic gastric microenvironment independent of body mass gain or microbiome changes.

## 1. Introduction

Gastric cancer (GC) accounts for approximately 10% of cancer-related deaths annually and is the second leading cause of cancer death worldwide [1]. *Helicobacter pylori* is the predominant cause of chronic inflammation and raises the risk of GC [2], and, in particular, is positively associated with gastric non-cardia adenocarcinoma [3]. However, only a small percentage of *H. pylori*-infected individuals ultimately develop GC [4,5]. Obesity is a critical risk factor for gastric cardia cancer, but not total GC [6]. It has been revealed that the obese population has a higher incidence of *H. pylori* infections [7], providing a possible mechanism for the observed morbidity of GC in obese individuals. While *H. pylori* infection is a known risk factor for developing GC, stimulation and modulation of the gastric mucosa via diet and resident microbiota could have important ramifications for human health.

Although several epidemiologic analyses have reported the causal relationship between dietary fat and the risk of GC, these studies are controversial because of locality, ethnicity, or no restricted region of GC (total, cardia, or antrum). Most dietary fats have been also reported to be associated with gastrointestinal tumors due to the induction of obesity [8,9]. However, some other studies report the inverse or are controversial [10,11]. Epidemiologic studies have also shown the association of dietary fat with gastric cancer [12,13]. Han et al. reported with meta-analysis that vegetable fat reduced GC risk, but animal fat did not [14]. We demonstrated that a high-fat diet (HFD) with lard induced intestinal metaplasia (which is the transformation of gastric epithelium into a type of intestinal-like epithelium) precancerous lesions of the stomach [15,16]. *H. felis*-induced gastric carcinogenesis is augmented in those fed with a lard diet [17]. In contrast, diets rich in olive oil are correlated with a lower incidence of gastric adenocarcinoma [18], and dietary cocoa ameliorates inflammation in diet-induced obese mice [19]. Thus, it is critically important to identify what kinds of dietary fats compel malignancy of gastric mucosa.

Nutrition can affect the microbial community in the intestine, and the microbiome contributes widely to systemic diseases [20,21]. Dysbiosis, a microbiome imbalance induced by HFD feeding is associated with gastrointestinal malignancy [22]. We have reported that a high lard diet induced intestinal metaplasia in the stomach [15] and severe dysbiosis with a larger dominance of the proportion of *Lactobacillus* [23] in accordance with pathogenesis changes in the stomach. There are several reports examining the different effects of dietary fat on intestinal microbiota in mice. Previous murine studies have shown that fish oil did not promote white adipose tissue inflammation through Toll-like receptor activation, whereas lard-fed mice did [24]. Enormous studies have shown microbiota changes due to various HFDs in the intestine, whereas few studies report on those in the stomach and their effects on gastric pathogenesis. 

Leptin is an adipocyte-derived hormone that critically regulates food intake and energy expenditure [25]. The stomach also produces leptin [26], and higher expression of gastric leptin and its receptor signaling results in the onset of gastric malignancy [27,28]. We have demonstrated that mice fed a HFD with lard (60% kcal fat) exhibit intestinal metaplasia [15] and an increase in stem cells and pluripotent gene expression mediated by leptin receptor signaling in the stomach [16]. In contrast, leptin-deficient *ob/ob* mice or leptin receptor mutated *db/db* mice, a type 2 diabetes model, are extraordinarily obese and do not show enhanced expression of stemness genes, such as Notch1 and Lgr5, and intracellular localization of β-catenin, when compared with HFD-fed wild-type (WT) mice [16]. Thus, we investigated a variety of diets that included animal and vegetable fats, and how each induced protumorigenicity in the stomach.

## 2. Materials and Methods

### 2.1. Animals, Diets, and Chemical

Male C57BL/6J (wild-type: WT) were studied at seven weeks of age (Japan SLC, Inc., Hamamatsu, Japan). The mice were housed individually in plastic cages at 24 °C ± 1 °C with lights on from 07:00 to 19:00. The mice were provided with either AIN-93M-based control-diet (CD) and diets with 60% of calories from fat containing lard (Lard), beef tallow (Beef), hydrogenated coconut oil (Coconut), linseed oil (Linseed), corn oil (Corn), olive oil (Olive), soybean oil (Soybean), and cocoa butter (Cocoa) (Oriental Yeast Japan, Table S1 and Figure S1) and water *ad libitum* for three months. To examine effect of HFDs in addition to the carcinogen, 1-methyl-3-nitro-1-nitrosoguanidine (MNNG; Tokyo Chemical Industry Co., Ltd., Tokyo, Japan), 100 mg/L MNNG in distilled water was provided to mice twice a week from five weeks of age. At seven weeks of age, in addition to MNNG administration, mice were fed CD or HFDs with Lard, Linseed, Olive, or Cocoa for three months. Animal care and experiments were conducted in accordance with the guidelines of the Prefectural University of Hiroshima Animal Care and Use Committee and Review Board (18A-008).

### 2.2. Histological Analysis

Paraffin-embedded sections of 10% formalin-fixed tissues were stained with either hematoxylin and eosin (H&E), or with periodic-acid Schiff (PAS) and Alcian blue. Immunofluorescence staining was performed after retrieval using a Retrievagen A kit (pH 6.0, BD Biosciences. San Jose, CA, USA), followed by overnight incubation with the primary Abs (Supplemental Table S2) and then reacted with Alexa Fluor 488 or Alexa Fluor 594-conjugated anti-rabbit, anti-mouse or anti-rat IgG Ab, as appropriate. Slides were mounted using ProLong Gold Antifade reagent with 4′,6-diamidino-2-phenylindole (DAPI; Life Technologies, Carlsbad, CA, USA). For alkaline phosphatase staining, after reacting with the primary Abs, slides were incubated with alkaline phosphatase-conjugated secondary antibody (Histofine Simple Stain AP, NICHIREI BIOSCIENCE INC., Tokyo, Japan). Alkaline phosphatase activity was measured using a substrate for alkaline phosphatase (ImmPact™ Vector Red, VECTOR Laboratories, Burlingame, CA, USA), and detected using a ZEISS Axio Imager 2 (Carl-Zeiss, Oberkochen, Germany). For each stomach specimen, all epithelial cells in randomly selected four high-power fields were evaluated for positivity of each stem cell marker. Three sections of the stomach from three CD- and HFDs-fed mice were evaluated. Quantitative analysis of the immunohistochemistry-stained sections was performed using ImageJ software v1.52 (National Institutes of Health, Bethesda, MD, USA). The assessment of mucosal alterations in the stomach was based on a summation of scores for hyperplasia (0, non-substantial alteration; 1, low; 2, moderate; and 3, high), cell infiltration (0, non-substantial alteration; 1, low; 2, moderate; and 3, high), loss of gastric glandular cells (0, non-substantial alteration; 4, low; 5, moderate; and 6, high), Alcian blue staining (0, nonsubstantial alteration; 4, focal; 5, diffuse; 6, very strong diffuse), parietal cell loss (0, nonsubstantial alteration; 4, moderate; and 5, high).

### 2.3. Plasma Assay

Serum was collected from blood obtained by cardiocentesis under anesthesia and stored at −80 °C until measurement. Leptin (Leptin ELISA, Millipore, St. Charles, MO, USA), insulin (Mouse Insulin ELISA kit, Shibayagi, Gunma, Japan), glucose (Glucose CII-test, Wako, Osaka, Japan), and non-esterified fatty acid (NEFA) (NEFA C-test, Wako) levels in the sera were measured according to the manufacturers’ protocols.

### 2.4. qPCR for Bacterial 16S rRNA Gene and Cytokine mRNA

DNA extraction from the gastrointestinal contents was performed using glass beads and phenol as described previously [23]. The mixture of glass beads and phenol was vortexed vigorously using a MicroSmash™ MS-100R system (Tomy Digital Biology, Tokyo, Japan) at 5000 rpm for 30 s. For detection and quantification of microbial number, qPCR analysis was carried out using 10-fold serially diluted extracted or standard DNA (kindly provided by Yakult Central Institute, Kunitachi, Japan) with group- and subgroup-specific primer sets in the KOD SYBR qPCR Mix (TOYOBO, Osaka, Japan) [23]. For detection of cytokine mRNA, total RNA from murine gastric mucosal tissue was extracted using RNeasy Mini Kits (QIAGEN, Valencia, CA, USA), according to the manufacturer’s protocols. cDNA was synthesized from gastric mucosal cells using the ReverTra Ace^®^ qPCR RT Kit (TOYOBO, Co., Ltd., Osaka, Japan) according to the manufacturer’s protocol. qRT-PCR was carried out using the KOD SYBR qPCR Mix (TOYOBO, Osaka, Japan) with specific primer sets (Table S3). And the products were detected on the AriaMx Real-Time PCR System (version 1.61, Agilent Technologies, Foster City, CA, USA). Relative changes in gene expression levels were calculated using the ΔΔCt method. 18S rRNA gene expression level was used for the normalization.

### 2.5. Statistical Analysis

Data are presented as the mean ± SD and were analyzed by ANOVA followed by the Holm–Sidak post-hoc test for multiple comparisons, and Pearson correlation analysis using Prism software version 6 (GraphPad, San Diego, CA, USA). A *p*-value less than 0.05 was considered statistically significant. 

## 3. Results

### 3.1. Obesity Does Not Necessarily Cause Intestinal Metaplasia in the Gastric Mucosa

**Figure 1:**
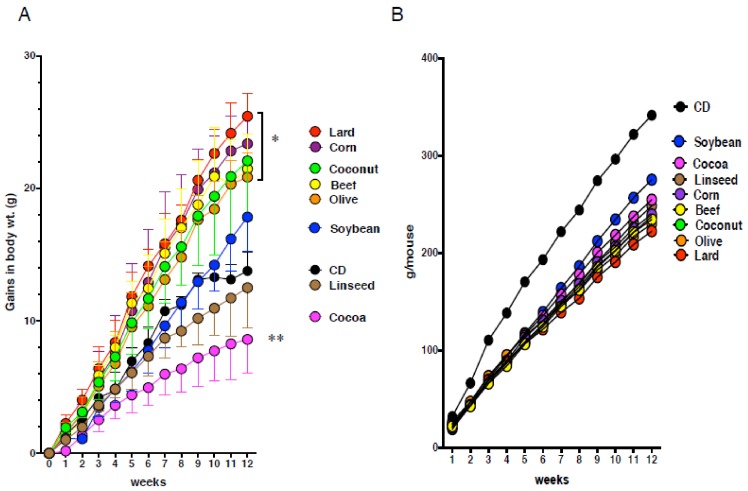
Alteration of body weight and food intake of C57BL/6J mice fed eight kinds of high-fat diets (HFDs). (**A**) Gains in body weight and (**B**) food intake of mice fed control (CD), beef tallow (Beef), Lard, hydrogenated coconut (Coconut), linseed oil (Linseed), corn oil (Corn), olive oil (Olive), soybean oil (Soybean), and cocoa butter (Cocoa) in C57BL/6J mice for 12 weeks. Data, shown as mean ± SD were analyzed by the one-way ANOVA followed by Holm-Sidak post-hoc test for multiple comparisons. * *p* < 0.01, ** *p* < 0.001 for HFDs versus CD. Eight mice per group were used.

**Figure 2:**
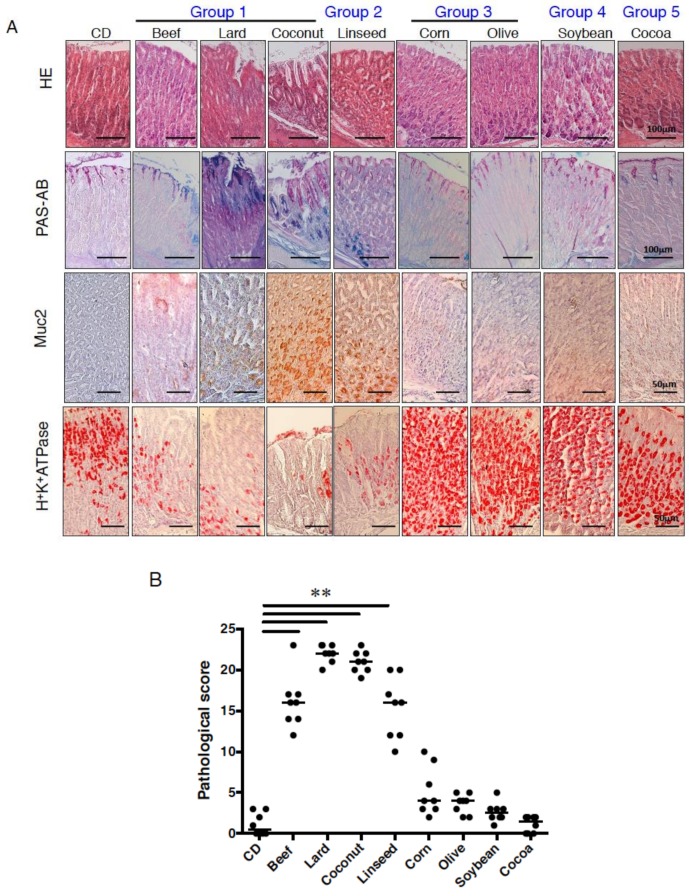
Pathological feature of gastric mucosa owing to various HFDs. (**A**) Gastric sections stained for periodic-acid Schiff (PAS)-Alcian blue, Muc2, and H^+^K^+^ATPase in mice fed experimental diets for 12 weeks. We utilized eight mice in each analysis, and representative data are shown. (**B**) The histological scores from the stomachs of mice fed CD or HFDs were graded according to the diagnostic criteria described in the Methods. Data presented as medians (99.9% confidence intervals) were analyzed by the one-way ANOVA followed by Holm-Sidak post-hoc test for multiple comparisons. ** *p* < 0.001. Eight mice per group were used.

To determine the effect of dietary fat on body weight and the pathogenesis of the gastric mucosa, C57BL/6J mice were fed eight kinds of HFDs (60% kcal from fat) or CD (9% kcal from fat) for three months (Table S1 and Figure S1). Mice fed lard (Lard), beef tallow (Beef), and hydrogenated coconut oil (Coconut) diets increased body weight as compared with CD-fed mice (Figure 1). These mice also exhibited increase in appearance of intestinal crypt-like structures concomitant with Alcian blue-positive cells, loss of parietal cells, which release gastric acid, which was supported by reduced expression of parietal cell-marker, H^+^K^+^ATPase, indicating that the feature of gastric epithelium was changed into that of intestinal epithelium (intestinal metaplasia) (Figure 2A). Mice fed linseed oil (Linseed) did not increase body weight, but clearly showed intestinal metaplasia (Figure 1 and Figure 2A). Corn and olive oil (Olive) diets induced an increase in body weight, but little expression of Muc2, and slightly increased H^+^K^+^ATPase expression. Mice fed a soybean oil (Soybean) diet showed no increase in body weight but did show the same alterations in the expression of the above markers. Mice fed cocoa butter (Cocoa) exhibited lower body weight compared with CD-fed mice, and similar mucosal morphology and features to CD-fed mice. Although the phenotype of the gastric pathology was different due to HFDs, food intake was similar (Figure 1). These alterations in pathogenesis were more prominently shown in gastric cardia than antrum. These results indicate that various dietary fats were divided into five groups in comparison to the CD group; increase in body weight and metaplasia onset (Lard, Beef, Coconut; group I), little increase in body weight but metaplasia onset (Linseed; group II), increase in body weigh without metaplasia onset (Corn, Olive; group III), neither increase in body weight nor metaplasia onset (Soybean; group IV), decreased body weight without metaplasia onset (Cocoa; group V) (Figure 1 and Figure 2B).

### 3.2. Intestinal Metaplasia in the Stomach Is Developed by HFDs that Induced Gastric Leptin Production 

**Figure 3:**
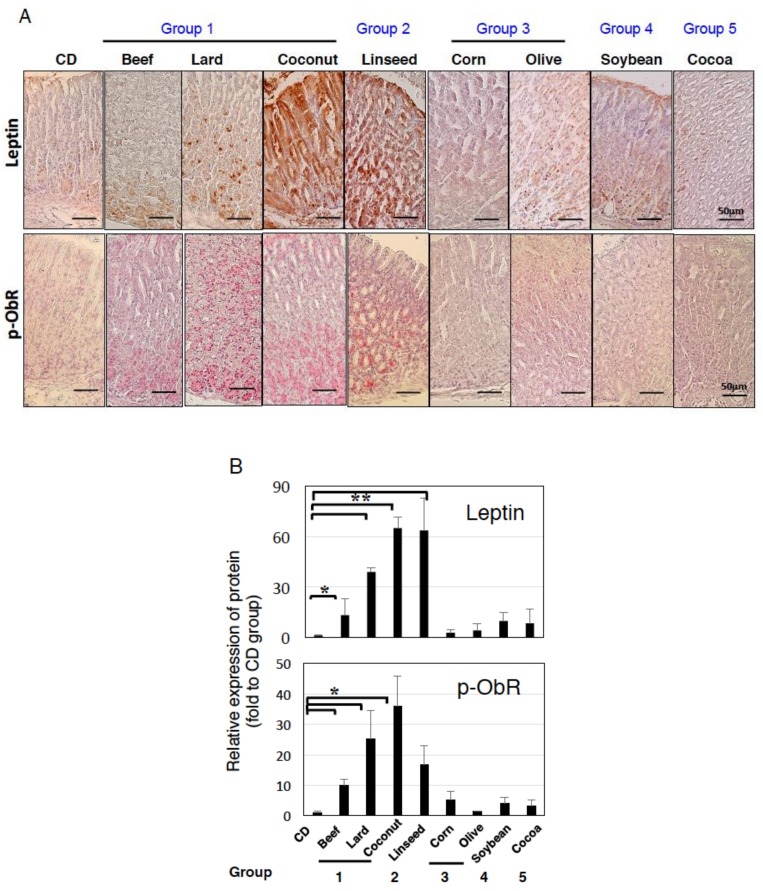
Difference of leptin and phosphorylated ObR expression in the stomach owing to HFDs feeding. (**A**) Gastric sections stained for leptin and phosphorylated ObR in mice fed experimental diets for 12 weeks. (**B**) Quantification of leptin and p-ObR expression shown in Figure 3 was performed by using ImageJ software described in the Materials and Methods. Data, shown as mean ± SD of eight mice were analyzed by the one-way ANOVA followed by Holm-Sidak post-hoc test for multiple comparisons. * *p* < 0.05, ** *p* < 0.001.

**Figure 4:**
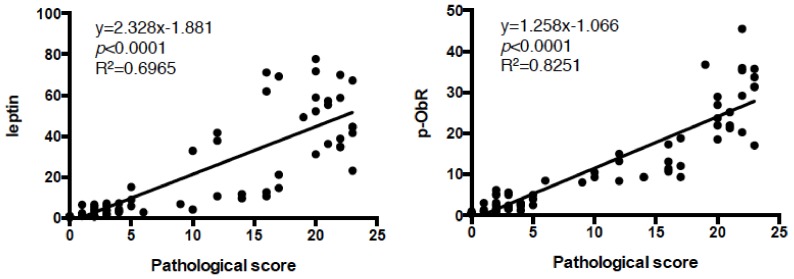
Highly correlation between pathogenesis and expression of leptin or p-ObR in the gastric mucosa of HFD-fed mice. Pearson correlation was performed for pathological score shown in Figure 2B and leptin or p-ObR expression shown in Figure 3B, and a significant correlation (*p* < 0.0001) was obtained.

**Figure 5:**
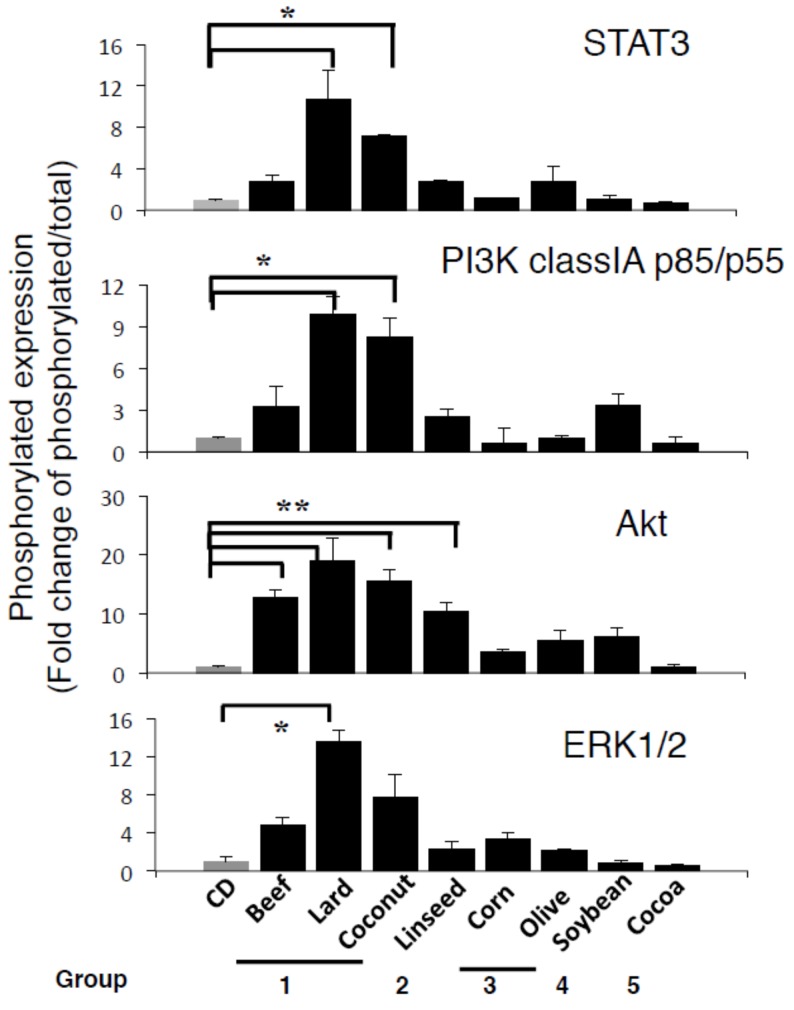
Upregulation of leptin receptor signaling in the gastric mucosa of group 1-type HFD-fed wild-type (WT) mice. Gastric sections stained for the phosphorylated STAT3, PI3K class I p85/p55, Akt, and ERK1/2, and total STAT3, PI3K class I p85/p55, Akt, and ERK2 in the gastric mucosa of WT fed for 12 weeks. Quantification of each expression level was performed by using ImageJ software described in the Methods. Data, shown as mean ± SD of eight mice were analyzed by the one-way ANOVA followed by Holm-Sidak post-hoc test for multiple comparisons. * *p* < 0.05, ** *p* < 0.01.

As we reported previously [15,16], high-lard diet feeding increased leptin and phosphorylated ObR expression in the stomach (Figure 3A,B). Beef also showed significant expression of leptin and p-ObR, and Coconut and Linseed diets further augmented their expression (Figure 3). In contrast, Corn, Olive, Soybean, and Cocoa diets did not. Phosphorylated ObR expression was shown in parallel with leptin expression (Figure 3A,B). Pathogenesis of intestinal metaplasia was strongly correlation to both expressions of leptin and p-ObR (Figure 4). Following leptin and phosphorylated ObR expression, STAT3, PI3K class I p85/p55, and ERK1/2 were also phosphorylated in the stomach of mice fed Lard, Coconut, and Linseed diets (Figure 5). In sera, the leptin concentration trended higher in those fed Lard, Coconut, and Corn but insulin, glucose, and NEFA concentration did not vary among HFDs (Figure S2). 

Chronic inflammatory mediators can be strongly associated with pleiotropic effects in the development of intestinal metaplasia and tumorigenesis. To identify potential inducers of intestinal metaplasia, we examined the mRNA expression of leptin, IL-11, and TNF-α in the gastric mucosa. Group I and II diets clearly increased leptin, in particular, Lard and Coconut also TNF-α level. IL-11 mRNA tended to increase in most HFDs except Cocoa diet. These results suggested that HFDs-accelerating the formation of intestinal metaplasia potently induced increased leptin expression and signaling in the stomach (Figure S3).

### 3.3. β- Catenin Signaling Is Increased in the Gastric Mucosa of Mice Fed HFDs Accelerating Gastric Leptin Production

**Figure 6:**
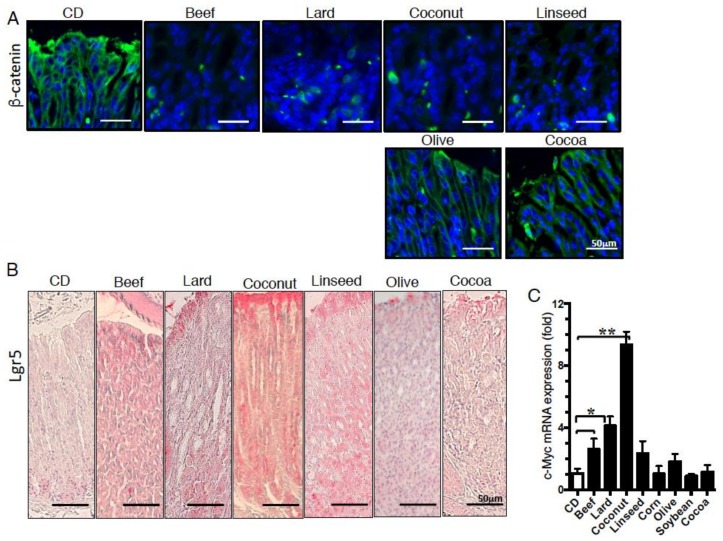
Difference in the intracellular and nuclear localization of β-catenin and the expression of β-catenin target molecules in the gastric mucosa of HFD-fed mice. Staining of the gastric mucosa with (**A**) β-catenin and (**B**) Lgr5 of mice fed experimental diets for 12 weeks. (**C**) mRNA expression of c-Myc of the gastric mucosa in mice fed experimental diets for 12 weeks. Data, shown as mean ± SD were analyzed by the one-way ANOVA followed by Holm-Sidak post-hoc test for multiple comparisons. * *p* < 0.05, ** *p* < 0.001. Eight mice per group were used.

We next examined β-catenin expression in the gastric mucosa of mice fed Lard, Linseed, Olive, or Cocoa diets, because PI3K plays a role in the stabilization of β-catenin via Akt activation [29]. As reported previously using Lard diet [16], Beef and Coconut also promotes intracellular or perinuclear localization of β-catenin. Furthermore, it was also observed in the stomach of Linseed diet-fed mice, whereas Olive and Cocoa suppressed these changes (Figure 6A). Moreover, Lgr5-positive cells, one of target molecules of β-catenin signaling, were increased concomitantly (Figure 6B). mRNA expression of c-Myc, which is a master regulator of cellular growth and metabolism [30], and target molecules of β-catenin signaling were also increased in gastric mucosa of Beef, Lard, and Coconut-fed mice (Figure 6C). These results indicate that HFDs of group I and II that induced intestinal metaplasia accelerate intracellular β-catenin expression and stem cell-like cells such as Lgr5, activated pluripotent molecules such as c-Myc, suggesting that pathogenesis of intestinal metaplasia depended on gastric leptin signaling mediated through β-catenin signaling.

### 3.4. Alteration of Microbial Component Induced by HFDs Is Irrespective of Body Mass

**Figure 7:**
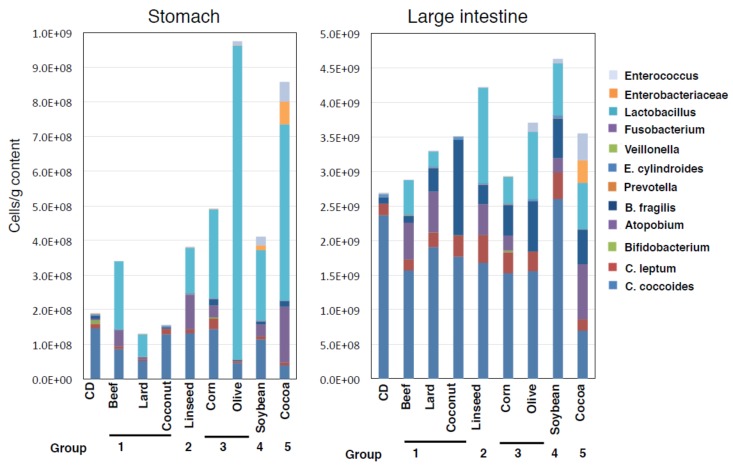
Alterations in microbial abundance in the gastrointestinal tract in various HFD-fed mice. Absolute numbers of the 12 major kinds of gastrointestinal bacteria in the stomach and large intestine, quantified at the group-specific level using qPCR. Data are represented as a stacked bar chart with eight mice per group.

We examined the abundance of 12 major kinds of gastrointestinal microbiota by using group-specific 16S rRNA-targeted primers. In the stomach, groups I and II diet-fed mice exhibited less alteration of total bacterial number, whereas Olive and Cocoa-fed mice showed increases (Figure 7). HFD-fed mice generally showed drastic decrease of *Bifidobacterium* occupancy (CD 6.1%, Beef 0.03%, Lard not detected, Coconut 0.03%, Linseed 0.05%, Corn 0.7%, Olive 0.0001%, Soybean 0.001%, and Cocoa 0.0004%) but trends in the increased total bacterial number with higher occupancy of *Lactobacillus*. This observation was not shown in Lard and Coconut diet group. In particular, Olive and Cocoa diets caused a much higher occupancy, 96% and 74%, respectively. Microbiota of large intestine also showed an increased occupancy with *Lactobacillus* though less and milder than that of the stomach. Linseed- and Soybean-fed mice exhibited slightly increased bacterial numbers. In both the stomach and large intestine, Cocoa-fed mice showed a preserved microbiome diversity. These results suggested that microbiome dysbiosis is not related with obesity or the onset of intestinal metaplasia of HFD-fed mice.

### 3.5. HFDs-Accelerating Gastric Leptin Production Enhance Dysplasia in Mice Administered MNNG

**Figure 8:**
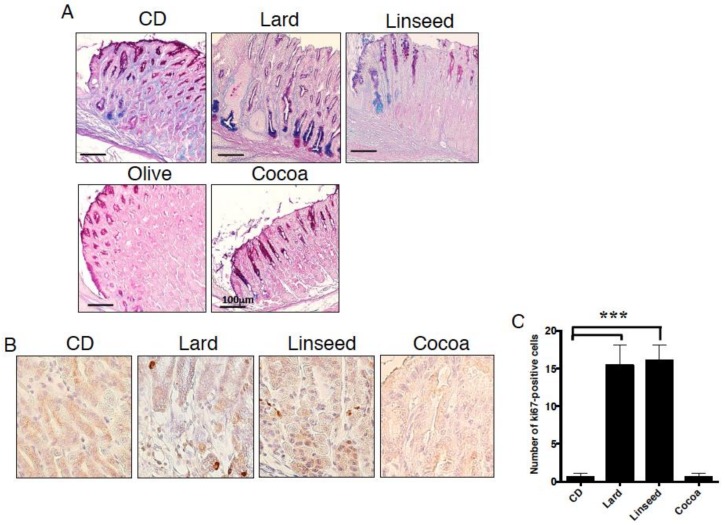
Acceleration of dysplasia in the stomach of the mice fed HFDs inducing gastric leptin. Gastric sections stained for (**A**) PAS-Alcian blue and (**B**) Ki67 in mice fed experimental diets for 12 weeks. Graphs next to each stained picture indicate numbers of positive cells in four randomly selected fields at 200× magnification in each group. Values represent the means ± SD. (**C**) Data, shown as mean ± SD were analyzed by the one-way ANOVA followed by Holm-Sidak post-hoc test for multiple comparisons. *** *p* < 0.0001. Five mice were used in each group.

To further investigate protumorigenicity of HFDs, we fed Lard, Linseed, Olive, and Cocoa diets to MNNG-administered mice. Since an administration of MNNG alone hardly induces gastric pathogenesis for short period [31], three month CD-fed mice had no tumors, but exhibited low-grade hyperplasia with Alcian blue-positive cells. In contrast, Lard-fed mice showed more prominent dysplasia and complete replacement of normal gastric glands to intestinal cryptic structure (Figure 8A). They also exhibited glandular metaplasia with many Alcian blue-positive cells at the basement of the foveola than the Lard-fed mice without MNNG (Figure 2A). In Linseed-fed mice, gastric mucosal structure was maintained, although there were increased Alcian blue-positive cells and decreased PAS-positive cells. The Olive diet induced hyperplasia and no intestinal type mucin. Cocoa-fed mice showed normal mucosal structure and expressed PAS-positive gastric type-mucus in spite of MNNG administration. We further examined cell proliferation in the gastric mucosa. In Lard or Linseed-fed mice, strong and high frequencies of Ki67-positive cells were detected from basal mucosa and weak positive cells spread across the mucosa in gastric cardia, whereas no such cells were observed in Cocoa diet group (Figure 8B,C). These results indicate that Lard and Linseed-type HFDs increased the tumorigenic environment in the stomach.

## 4. Discussion

This study presents the first evidence that HFD consumption does not necessarily induce an obesity-associated protumorigenic environment in the stomach that is accelerated by HFDs, actively stimulating gastric leptin production. We further showed that microbiome changes are not necessary for obesity and the onset of intestinal metaplasia in the stomach. Links between obesity and microbial community alteration have been considered as a cause gastrointestinal malignancy. However, our findings highlight that body mass increase is independent of pathogenesis and dysbiosis in the stomach. Thus, these results indicate that, irrespective of the induction of obesity or dysbiosis, HFDs elicit a gastric leptin-accelerated protumorigenic milieu in the stomach. These findings greatly contribute to improving the current understanding of dietary fat for the prevention of GC. 

We compared eight kinds of HFDs adjusted to 60% kcal from fat. In spite of the fact that food intake was similar among all HFDs, body weight gains were different. Increased body mass can be caused by the digestibility and absorption of fat. Cocoa butter has been known to be a low-energy fat. In general, lipid digestibility is less than 95%. In contrast, cocoa butter digestibility is 60–70%, as the binding of calcium to form poorly digested fatty acid-calcium complexes results in low absorption [32,33]. Other than digestibility and absorption, the type of fatty acids consumed is also a critical factor to body mass and pathogenesis. Fatty acids modulate mucosal cell growth and DNA damage [34] and contribute to the initiation and progression in various cancers [35,36,37]. Palmitic acid and saturated fatty acids promote metastasis of GC cells via fatty acid-binding protein 5 cascade [38]. Oleic acid enhances the invasiveness of GC cells via PI3K-Akt pathway [39]. N-3 polyunsaturated fatty acids have the ability to suppress pro-inflammatory cytokine production in GC patients [40]. However, our study revealed that the pattern of fatty acids might not be independent of metaplasia induced. The group I diet induced metaplasia, and while the Lard and Beef diets contain similar palmitic and oleic acid-rich fatty acids components, Coconut diet contains lauric acid-rich fatty acids (Figure S1). Hydrogenated vegetable oils are *trans* saturated fatty acids with an increased melting point and were established as alternatives to lard and butter in processed foods. While *trans* fatty acids have been reported to be associated with health disorders such as diabetes, cardiovascular disease, obesity, and cancers such as mammary and prostatic [41], no report indicates the gastrointestinal tumorigenic effects. Cocoa butter is composed of similar fatty acids as lard and beef tallow, however, the mice fed Cocoa diet kept lean with a healthy gastric mucosa. Moreover, dietary cocoa ameliorates the inflammatory mediators, IL-6 and MCP-1, in diet-induced obese mice [19] and inhibits colorectal cancer induced by AOM/DSS [42]. The Mediterranean diet, characterized by increased consumption of olive oil, could prevent gastric cancer [18], although Olive-fed mice become obese as shown in our study. There are several factors of fatty acid combination, digestibility, and absorption that influence pathogenesis, thus, it will be necessary to investigate what factors are involved in HFD consumption influencing the stemness and protumorigenesis of the stomach. 

It has not been investigated clearly how dietary fat modulates intracellular signaling for cell proliferation and protumorigenicity. β-catenin is a proto-oncogene and its signaling induces cell proliferation, differentiation, and survival [43,44]. In this study, in addition to Lard, we found that Beef, Coconut, and Linseed (groups I and II) also accelerated gastric leptin production irrespective of body mass and caused intestinal metaplasia. In mice fed group I and II diets, increased phosphorylation of STAT3 and p85/p55 PI3K class IA, and the nuclear localization and intracellular accumulation of β-catenin, and the induction of Lgr5 and c-Myc induction was clearly shown, while no changes were seen in the group III-, IV-, and V-fed mice. In addition, it has been reported the Cocoa diet suppressed permeability of intestine by sustained occludin and ZO-1, tight junction molecules used for the maintenance of intestinal epithelial barrier [45]. Through these mechanisms, it can support mucosal integrity and suppress dysregulation of stemness. This study strongly suggested that gastric leptin acts independently of the physiological role in appetite suppression and energy expenditure and affects tumorigenesis in the stomach.

Intestinal microbiota has been considered to have a potential role in obesity and gastrointestinal malignancies [46]. Microbiome imbalance, dysbiosis has been considered to increase the development of inflammation and cancer. Although the occurrence of a high ratio of Firmicutes/Bacteroidetes has been shown in the intestinal feces in both obese animals and humans [21,47,48], recently this finding is controversial [49,50]. Very recently, we found a considerable abundance of gastric microbiota in mice, although the gastric environment has been considered to be unfavorable for bacterial growth due to lower pH, and showed *Lactobacillus* dominance in intestinal metaplasia in the stomach of mice fed high lard diet [23]. Mice fed group I and II diets showed similar bacterial number to CD, however, the mice had comparatively lower number than group III, IV, and V diets. While dysbiosis enriched *Lactobacillus* population in the stomach of chronic *H. pylori*-infected patients under the reduced gastric acid secretion conditions [51], dysbiosis was not associated with onset of intestinal metaplasia in this study. All HFDs tended to increase microbial number and higher occupancy of *Lactobacillus* in the stomach. These effects were most pronounced in the Olive and Cocoa groups, in which these diets failed onset of intestinal metaplasia. Furthermore, in Cocoa-fed mice, the microbial community in both the stomach and large intestine tended to increase in diversity more than the other diet groups. We have reported that leptin signaling shapes the microbe community to collectively modulate the progression of pathogenesis in the stomach [23]. It will be necessary to investigate the significance of body mass, dysbiosis, and the pathogenesis in the gastric mucosa mediated by leptin signaling in the stomach to provide tumorigenesis or healthy in the gastric environment.

In conclusion, we demonstrated that intestinal metaplasia, which shapes the microbial community to collectively modulate pathological progression in the stomach, is independent of body mass and the microbiota community composition and depends on HFD-induced gastric leptin signaling. Although we demonstrated differences in dietary fat with respect to the acceleration or suppression of intestinal metaplasia in mice, further studies are needed to clarify precisely how dietary fat-induced gastric leptin affects cell development or the protumorigenic environment in the stomach. Our findings may suggest novel targets for the development of preventive strategies of dietary life against GC, especially in the context of obesity.

## Supplementary Materials

The following are available online at https://www.mdpi.com/2072-6643/11/9/2127/s1, Figure S1. Fatty acid composition of beef tallow (Beef), Lard, hydrogenated coconut oil (Coconut), linseed oil (Linseed), corn oil (Corn), olive oil (Olive), soybean oil (Soybean), and cocoa butter (Cocoa), Figure S2. Alteration of index associated with obesity. Serum leptin, insulin, glucose, and NEFA in mice fed experimental diets for 12 weeks. Data, shown as mean ± SD were analyzed by the one-way ANOVA followed by Holm-Sidak post-hoc test for multiple comparisons. Figure S3. Augmented mRNA expression of leptin in Lard and Linseed-type diets. Data, shown as mean ± SD of 8 mice were analyzed by the one-way ANOVA followed by Holm-Sidak post-hoc test for multiple comparisons. * *p* < 0.05, ** *p* < 0.001. Table S1. Diet composition, Table S2. Antibody list: Antibodies used for immunohistochemistry, Table S3. Primers used for qPCR.

## References

[1] Torre L.A., Bray F., Siegel R.L., Ferlay J., Lortet-Tieulent J., Jemal A. (2015). Global cancer statistics, 2012. CA Cancer J. Clin..

[2] Bhandari A., Crowe S.E. (2012). Helicobacter pylori in gastric malignancies. Curr. Gastroenterol. Rep..

[3] Helicobacter and Cancer Collaborative Group (2001). Gastric cancer and Helicobacter pylori: A combined analysis of 12 case control studies nested within prospective cohorts. Gut.

[4] Peek R.M., Crabtree J.E. (2006). Helicobacter infection and gastric neoplasia. J. Pathol..

[5] Wroblewski L.E., Peek R.M., Wilson K.T. (2010). Helicobacter pylori and gastric cancer: Factors that modulate disease risk. Clin. Microbiol. Rev..

[6] Chen Y., Liu L., Wang X., Wang J., Yan Z., Cheng J., Gong G., Li G. (2013). Body mass index and risk of gastric cancer: A meta-analysis of a population with more than ten million from 24 prospective studies. Cancer Epidemiol. Biomark. Prev..

[7] Dutta S.K., Arora M., Kireet A., Bashandy H., Gandsas A. (2009). Upper gastrointestinal symptoms and associated disorders in morbidly obese patients: A prospective study. Dig. Dis. Sci..

[8] O’Keefe S.J. (2016). Diet, microorganisms and their metabolites, and colon cancer. Nat. Rev. Gastroenterol. Hepatol..

[9] Butler L.M., Wang R., Koh W.P., Stern M.C., Yuan J.M., Yu M.C. (2009). Marine n-3 and saturated fatty acids in relation to risk of colorectal cancer in Singapore Chinese: A prospective study. Int. J. Cancer.

[10] Liu L., Zhuang W., Wang R.Q., Mukherjee R., Xiao S.M., Chen Z., Wu X.T., Zhou Y., Zhang H.Y. (2011). Is dietary fat associated with the risk of colorectal cancer? A meta-analysis of 13 prospective cohort studies. Eur. J. Nutr..

[11] O’Doherty M.G., Freedman N.D., Hollenbeck A.R., Schatzkin A., Murray L.J., Cantwell M.M., Abnet C.C. (2012). Association of dietary fat intakes with risk of esophageal and gastric cancer in the NIH-AARP diet and health study. Int. J. Cancer.

[12] Graham S., Haughey B., Marshall J., Brasure J., Zielezny M., Freudenheim J., West D., Nolan J., Wilkinson G. (1990). Diet in the epidemiology of gastric cancer. Nutr. Cancer.

[13] Hu J., La Vecchia C., Negri E., de Groh M., Morrison H., Mery L., Canadian Cancer Registries Epidemiology Research G. (2015). Macronutrient intake and stomach cancer. Cancer Causes Control..

[14] Han J., Jiang Y., Liu X., Meng Q., Xi Q., Zhuang Q., Han Y., Gao Y., Ding Q., Wu G. (2015). Dietary Fat Intake and Risk of Gastric Cancer: A Meta-Analysis of Observational Studies. PLoS ONE.

[15] Inagaki-Ohara K., Okamoto S., Takagi K., Saito K., Arita S., Tang L., Hori T., Kataoka H., Matsumoto S., Minokoshi Y. (2016). Leptin receptor signaling is required for high-fat diet-induced atrophic gastritis in mice. Nutr. Metab..

[16] Arita S., Kinoshita Y., Ushida K., Enomoto A., Inagaki-Ohara K. (2016). High-fat diet feeding promotes stemness and precancerous changes in murine gastric mucosa mediated by leptin receptor signaling pathway. Arch. Biochem. Biophys..

[17] Ericksen R.E., Rose S., Westphalen C.B., Shibata W., Muthupalani S., Tailor Y., Friedman R.A., Han W., Fox J.G., Ferrante A.W. (2014). Obesity accelerates Helicobacter felis-induced gastric carcinogenesis by enhancing immature myeloid cell trafficking and TH17 response. Gut.

[18] Castello A., Fernandez de Larrea N., Martin V., Davila-Batista V., Boldo E., Guevara M., Moreno V., Castano-Vinyals G., Gomez-Acebo I., Fernandez-Tardon G. (2018). High adherence to the Western, Prudent, and Mediterranean dietary patterns and risk of gastric adenocarcinoma: MCC-Spain study. Gastric Cancer.

[19] Gu Y., Yu S., Lambert J.D. (2014). Dietary cocoa ameliorates obesity-related inflammation in high fat-fed mice. Eur. J. Nutr..

[20] Sittipo P., Lobionda S., Lee Y.K., Maynard C.L. (2018). Intestinal microbiota and the immune system in metabolic diseases. J. Microbiol..

[21] Ley R.E., Backhed F., Turnbaugh P., Lozupone C.A., Knight R.D., Gordon J.I. (2005). Obesity alters gut microbial ecology. Proc. Natl. Acad Sci. USA.

[22] Carding S., Verbeke K., Vipond D.T., Corfe B.M., Owen L.J. (2015). Dysbiosis of the gut microbiota in disease. Microb. Ecol. Health Dis..

[23] Arita S., Inagaki-Ohara K. (2019). High-fat diet-induced modulations of leptin signaling and gastric microbiota drive precancerous lesions in the stomach. Nutrition.

[24] Caesar R., Tremaroli V., Kovatcheva-Datchary P., Cani P.D., Backhed F. (2015). Crosstalk between Gut Microbiota and Dietary Lipids Aggravates WAT Inflammation through TLR Signaling. Cell Metab..

[25] Friedman J.M., Halaas J.L. (1998). Leptin and the regulation of body weight in mammals. Nature.

[26] Bado A., Levasseur S., Attoub S., Kermorgant S., Laigneau J.P., Bortoluzzi M.N., Moizo L., Lehy T., Guerre-Millo M., Le Marchand-Brustel Y. (1998). The stomach is a source of leptin. Nature.

[27] Howard J.M., Pidgeon G.P., Reynolds J.V. (2010). Leptin and gastro-intestinal malignancies. Obes. Rev..

[28] Inagaki-Ohara K. (2019). Gastric Leptin and Tumorigenesis: Beyond Obesity. Int. J. Mol. Sci..

[29] Lovatt M., Bijlmakers M.J. (2010). Stabilisation of beta-catenin downstream of T cell receptor signalling. PLoS ONE.

[30] Miller D.M., Thomas S.D., Islam A., Muench D., Sedoris K. (2012). c-Myc and cancer metabolism. Clin. Cancer Res..

[31] Huang L., Qi D.J., He W., Xu A.M. (2017). Omeprazole promotes carcinogenesis of fore-stomach in mice with co-stimulation of nitrosamine. Oncotarget.

[32] Chen I.S., Subramaniam S., Vahouny G.V., Cassidy M.M., Ikeda I., Kritchevsky D. (1989). A comparison of the digestion and absorption of cocoa butter and palm kernel oil and their effects on cholesterol absorption in rats. J. Nutr..

[33] Apgar J.L., Shively C.A., Tarka S.M. (1987). Digestibility of cocoa butter and corn oil and their influence on fatty acid distribution in rats. J. Nutr..

[34] Zeng L., Wu G.Z., Goh K.J., Lee Y.M., Ng C.C., You A.B., Wang J., Jia D., Hao A., Yu Q. (2008). Saturated fatty acids modulate cell response to DNA damage: Implication for their role in tumorigenesis. PLoS ONE.

[35] Camarda R., Zhou A.Y., Kohnz R.A., Balakrishnan S., Mahieu C., Anderton B., Eyob H., Kajimura S., Tward A., Krings G. (2016). Inhibition of fatty acid oxidation as a therapy for MYC-overexpressing triple-negative breast cancer. Nat. Med..

[36] Hashimoto S., Mikami S., Sugino H., Yoshikawa A., Hashimoto A., Onodera Y., Furukawa S., Handa H., Oikawa T., Okada Y. (2016). Lysophosphatidic acid activates Arf6 to promote the mesenchymal malignancy of renal cancer. Nat. Commun..

[37] Jeon S.M., Chandel N.S., Hay N. (2012). AMPK regulates NADPH homeostasis to promote tumour cell survival during energy stress. Nature.

[38] Pan J., Dai Q., Zhang T., Li C. (2019). Palmitate acid promotes gastric cancer metastasis via FABP5/SP1/UCA1 pathway. Cancer Cell Int..

[39] Xiang F., Wu K., Liu Y., Shi L., Wang D., Li G., Tao K., Wang G. (2017). Omental adipocytes enhance the invasiveness of gastric cancer cells by oleic acid-induced activation of the PI3K-Akt signaling pathway. Int. J. Biochem. Cell Biol..

[40] Mocellin M.C., Fernandes R., Chagas T.R., Trindade E. (2018). A meta-analysis of n-3 polyunsaturated fatty acids effects on circulating acute-phase protein and cytokines in gastric cancer. Clin. Nutr..

[41] Ali Abd El-Aal Y., Mohamed Abdel-Fattah D., El-Dawy Ahmed K. (2019). Some biochemical studies on trans fatty acid-containing diet. Diabetes Metab. Syndr..

[42] Saadatdoust Z., Pandurangan A.K., Ananda Sadagopan S.K., Mohd Esa N., Ismail A., Mustafa M.R. (2015). Dietary cocoa inhibits colitis associated cancer: A crucial involvement of the IL-6/STAT3 pathway. J. Nutr. Biochem..

[43] Blanpain C., Fuchs E. (2009). Epidermal homeostasis: A balancing act of stem cells in the skin. Nat. Rev. Mol. Cell Biol..

[44] Clevers H., Nusse R. (2012). Wnt/beta-catenin signaling and disease. Cell.

[45] Zhong W., Li Q., Xie G., Sun X., Tan X., Sun X., Jia W., Zhou Z. (2013). Dietary fat sources differentially modulate intestinal barrier and hepatic inflammation in alcohol-induced liver injury in rats. Am. J. Physiol. Gastrointest. Liver Physiol..

[46] Vivarelli S., Salemi R., Candido S., Falzone L., Santagati M., Stefani S., Torino F., Banna G.L., Tonini G., Libra M. (2019). Gut Microbiota and Cancer: From Pathogenesis to Therapy. Cancers.

[47] Turnbaugh P.J., Hamady M., Yatsunenko T., Cantarel B.L., Duncan A., Ley R.E., Sogin M.L., Jones W.J., Roe B.A., Affourtit J.P. (2009). A core gut microbiome in obese and lean twins. Nature.

[48] Ferrer M., Ruiz A., Lanza F., Haange S.B., Oberbach A., Till H., Bargiela R., Campoy C., Segura M.T., Richter M. (2013). Microbiota from the distal guts of lean and obese adolescents exhibit partial functional redundancy besides clear differences in community structure. Environ. Microbiol..

[49] Arumugam M., Raes J., Pelletier E., Le Paslier D., Yamada T., Mende D.R., Fernandes G.R., Tap J., Bruls T., Batto J.M. (2011). Enterotypes of the human gut microbiome. Nature.

[50] Patil D.P., Dhotre D.P., Chavan S.G., Sultan A., Jain D.S., Lanjekar V.B., Gangawani J., Shah P.S., Todkar J.S., Shah S. (2012). Molecular analysis of gut microbiota in obesity among Indian individuals. J. Biosci..

[51] Ferreira R.M., Pereira-Marques J., Pinto-Ribeiro I., Costa J.L., Carneiro F., Machado J.C., Figueiredo C. (2018). Gastric microbial community profiling reveals a dysbiotic cancer-associated microbiota. Gut.

