# Supplementary material for: Dietary Fat-Accelerating Leptin Signaling Promotes Protumorigenic Gastric Environment in Mice
Source: Nutrients. 2019 Sep 6;11(9):2127. doi: 10.3390/nu11092127 (PMC6770546; doi:10.3390/nu11092127)
Supplement: Supplementary file 1 [file nutrients-11-02127-s001.pdf]

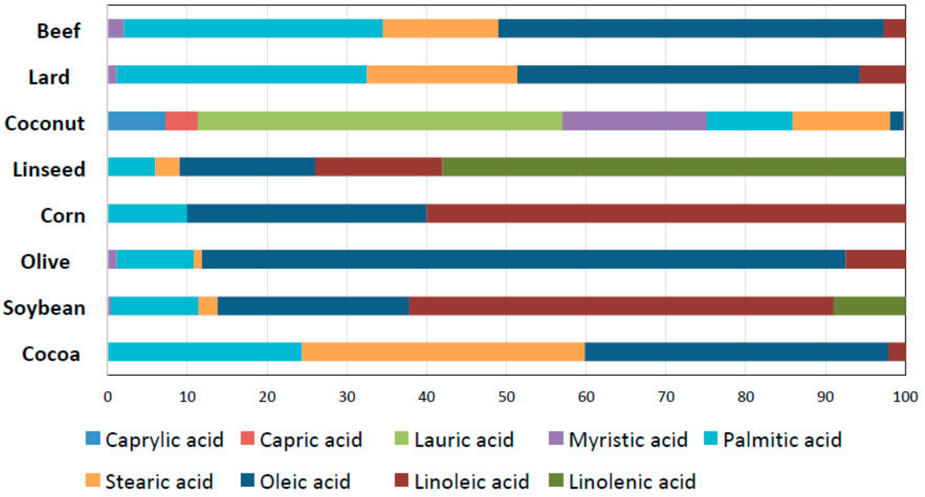

Figure S1. Fatty acid composition of beef tallow (Beef), Lard, hydrogenated coconut oil (Coconut), linseed oil (Linseed), corn oil (Corn), olive oil (Olive), soybean oil (Soybean), and cocoa butter (Cocoa).

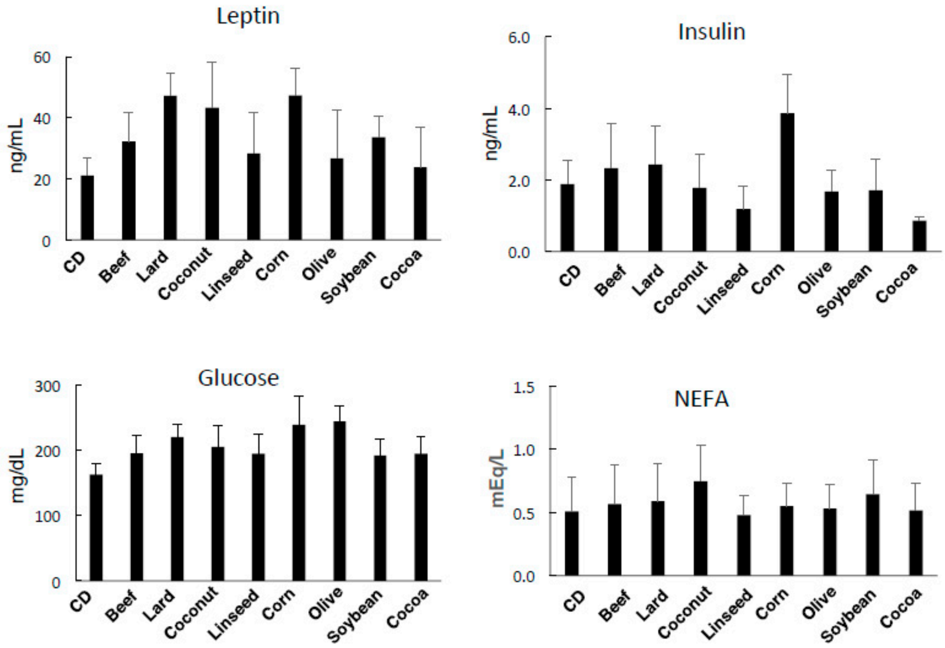

Figure S2. Alteration of index associated with obesity. Serum leptin, insulin, glucose, and NEFA in mice fed experimental diets for 12 weeks. Data, shown as mean ± SD were analyzed by the one-way ANOVA followed by Holm-Sidak post-hoc test for multiple comparisons.

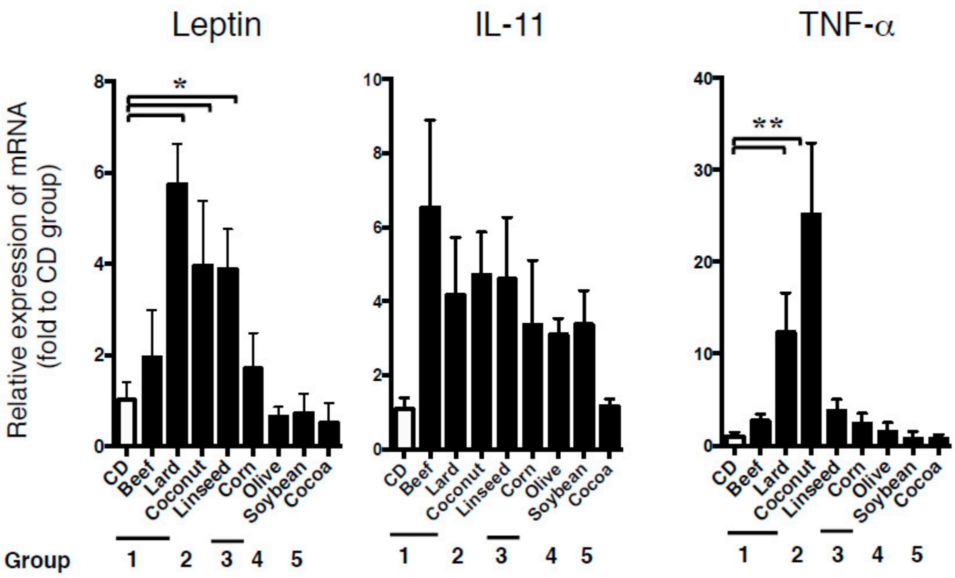

Figure S3. Augmented mRNA expression of leptin in Lard and Linseed-type diets. Data, shown as mean ± SD of 8 mice were analyzed by the one-way ANOVA followed by Holm-Sidak post-hoc test for multiple comparisons. * $p < 0.05$, ** $p < 0.001$.

Table S1 Diet composition.

Ingredient (g/100g)	CD	HFDs
Casein	14.00	14.00
L-Cystine	0.18	0.18
a-Corn starch	54.97	22.97
Maltodextrin	9.00	9.00
Sucrose	8.10	8.10
Soybean oil	4.00	4.00
Cellulose	5.00	5.00
AIN-93M mineral mix	3.50	3.50
AIN-93 vitamin mix	1.00	1.00
Choline bitartrate	0.25	0.25
tert-Butylhydroquinone	0.0008	0.0008
Fats (Lard, Beef, Coconut, Linseed, Corn, Olive, Soybean, Cocoa	0.00	32.00

Table S2 Antibody list: Antibodies used for immunohistochemistry.

Peptide/protein target	Manufacturer	Species raised in; monoclonal or polyclonal
Leptin	Abcam (ab16227)	Rabbit polyclonal
p-ObR (Tyr 1138)	Santa Cruz Biotech (sc-16421)	Rabbit polyclonal
Muc2	Santa Cruz Biotech (sc-15334)	Rabbit polyclonal
Lgr5	Abcam (ab75732)	Rabbit polyclonal
H^+K^+ ATPase	Abcam (ab 2866)	Mouse monoclonal
p-STAT3 (Tyr705)	Cell Signaling (9145S)	Rabbit polyclonal
STAT3	Santa Cruz Biotech (sc-8019)	Mouse monoclonal
p-p44/p42 MAPK (ERK1/2)	Cell Signaling (9106S)	Mouse monoclonal
MAPK (ERK2)	Santa Cruz (sc-1647)	Mouse monoclonal
p-PI3K p85/p55	Cell Signaling (#4228)	Rabbit polyclonal
PI3 Kinase p85	Cell Signaling (#4257)	Rabbit polyclonal
p-Akt (Ser 473)	Cell Signaling (#4060)	Rabbit polyclonal
Akt	Cell Signaling (#4691)	Rabbit polyclonal
β-catenin	Abcam (ab227499)	Rabbit polyclonal
Ki67	Abcam (ab15580)	Rabbit polyclonal

Table S3 Primers used for qPCR.

Name		Primer sequences	Accession Number
Lept	sense	5'-TGA CAC CAA AAC CCT CAT CA-3'	NM_008493.3
	anti-sense	5'-TCA TTG GCT ATC TGC AGC AC-3'	
Il-11	sense	5'- CTC TTC AGA CCC TCG AGC AG-3'	NM_008350.4
	anti-sense	5'- AAG CTG CAA AGA TCC CAA TG-3'	
Tnf-a	sense	5'-CGT CAG CCG ATT TGC TAT CT-3'	NM_001278601.1
	anti-sense	5'-CGG ACT CCG CAA AGT CTA AG-3'	
18S	sense	5′-GTA ACC CGT TGA ACC CCA TT-3′	NM_003278.3
	anti-sense	5′-CCA TCC AAT CGG TAG TAG CG -3′	
